# Experiences Among Health Care Personnel with Remote General Movement Assessment for the Prediction of Cerebral Palsy in High-Risk Infants

**DOI:** 10.3390/jcm14207390

**Published:** 2025-10-19

**Authors:** Wenche Ann Similä, Lars Adde

**Affiliations:** 1 Department of Public Health and Nursing, Norwegian University of Science and Technology, 7049 Trondheim, Norway; 2 Department of Clinical and Molecular Medicine, Norwegian University of Science and Technology, 7049 Trondheim, Norway; 3 Clinic of Rehabilitation, St. Olavs Hospital, Trondheim University Hospital, 7006 Trondheim, Norway

**Keywords:** general movement assessment, early predictive assessment, cerebral palsy, infant, parent, health care personnel, video recording, clinical practice, qualitative research

## Abstract

**Background/Objectives**: General movement assessment (GMA) is a clinical assessment tool used to predict risk for cerebral palsy (CP) in young infants. Equal access is challenging since GMA-trained personnel is a limited resource. An implementation study aimed to offer all high-risk infants born in the Central Norway Regional Health Authority equal access to GMA as part of the standard follow-up. This study explored the local health care personnel (HCP) experiences with early risk assessment for CP in young infants using remote GMA. **Methods**: This was a qualitative study with one focus group and four individual interviews. Participants were HCP from the local follow-up clinics who had experience with GMA. Analyses were inspired by Malterud’s systematic text condensation. **Results**: Attitudes towards GMA were, in general, positive, and GMA was considered an important and gentle examination contributing to earlier initiation of correct follow-up actions and appropriate treatment. The GMA results could improve communication between HCP and parents, and lead to a closer local municipality follow-up if GMA result was abnormal. Parents were given an active role with home video recording, which was considered family empowering. Especially pediatricians wanted more detailed information about the qualities of spontaneous movements to support clinical decision-making. **Conclusions**: This study indicated that further implementation of the GMA method to assess the risk of CP in high-risk infants could be recommended, and that GMA was a gentle method for the purpose. As suggested by pediatricians in this study, more detailed assessments using the GMA beyond FMs could be further explored as support to clinical decisions. The insight from this study may inform implementation in similar contexts.

## 1. Introduction

The General Movement Assessment (GMA) is the single most predictive clinical tool for cerebral palsy (CP) and is recommended for clinical use in the follow-up of high-risk infants [1,2,3]. The GMA is based on expert observations of the fidgety type of infant general movements (GMs) in 3–5 min long video recordings between 12 and 17 weeks post-term age [3,4]. GMA by trained observers can use both home- and hospital-based videos, providing access to early risk assessment where local GMA expertise is not available [5,6]. To facilitate the involvement of parents, reduce travel costs to hospitals, and optimize the quality of infants’ states in video recordings, smartphone apps for home video recordings have been developed. The Neuro Motion, Baby Moves, and In-Motion apps have been used successfully for recording of GMs at home [7,8,9]. Parents perform video recording with high engagement, and it is shown that there are few worries about using the GMA [2,7]. Recent studies show that implementation of remote GMA in clinical follow-up programs is possible [2,7,10] and that home-based videos are feasible for remote GMA [6,7]. This enables remote GMA at centralized hospital centers with access to trained and experienced GMA observers [2,10,11,12].

Equal access to early predictive evaluation methods has the potential to improve the quality of standardized follow-up and, consequently, improve access to local municipality health services for infants in need of early intervention [2]. It is also important to reassure families with infants who are unlikely to develop CP.

Although implementation studies using GMA are promising [2,10], no previous study has explored how early risk assessment by remote GMA is perceived and utilized by health care personnel (HCP) in hospital-based follow-up programs. Furthermore, the potential implications for follow-up both at the hospital and in the local municipality remain previously unexplored. Despite the large numbers of clinical studies using GMA over the last two decades, we know little about how GMA results influence medical decision-making and further follow-up.

The primary aim of this study was therefore to explore the experiences among HCP in hospital-based follow-up programs with early risk assessment for CP using remote GMA. Explored experiences in this study included how GMA results were documented, read, and understood by HCP, and how GMA results influenced decisions on further follow-up. Additionally, HCP experiences with the video recordings of infants in hospitals and parents’ home video recording were explored through the interviews.

## 2. Materials and Methods

This was a qualitative study with one focus group and four individual interviews conducted between February and March 2023.

The general movement assessment (GMA) procedure and clinical study setting were that remote GMA was offered to families of infants referred to neurodevelopmental follow-up programs at discharge from the neonatal intensive care unit (NICU) across three sites (St. Olavs hospital in Trondheim, Ålesund hospital, and Levanger Hospital) in the Central Norway Regional Health Authority. Two home video recordings were scheduled during the fidgety movements (FM) period at 12+1 to 14+6 and 15+1 to 17+6 weeks post-term age. A parallel video recording was made at an in-hospital appointment between 12+1 and 17+6 weeks post-term age. This was to ensure a video recording if parents did not transfer any home-based video recording during the appropriate time window.

GMA experts assessed and documented the results. All the video recordings were transferred to a central GMA team located in one of the hospitals. This team, comprising three certified and experienced GMA assessors, received all videos and conveyed results to the relevant local follow-up team by documenting GMA results in a regional electronic health record system. The GMA result of each individual film was documented with a conclusion indicating “at risk for cerebral palsy (CP)” (absence of fidgety movements (FM-)), “low risk for CP” (presence of fidgety movements (FM+)), or “uncertain GMA” (FM sporadic or FM exaggerated). Those with an uncertain result received a recommendation to make an additional video. Further follow-up was planned at the discretion of the local follow-up team, consisting of pediatricians, physical therapists, and nurses at each hospital site. For more details about the implementation project, see Adde et al. [2].

We invited all health care personnel (HCP) who had participated in the implementation study of remote GMA in the Central Norway Regional Health Authority [2] for this qualitative study. The first author carried out the recruitment. Invitations were sent by e-mail to ten HCP, including pediatricians, physical therapists, and a nurse. All three hospitals were represented in the interviews. Three HCP volunteered for individual interviews, and five HCP volunteered for participation in a focus group interview. Both pediatricians and physical therapists were represented. One was prevented from participating in the focus group interview and was individually interviewed on a later occasion. The participants signed an informed consent.

Reflexivity was important for the protection of privacy for the participants. Especially, privacy and volunteering were considered carefully, since there were few potential HCP eligible to participate. Therefore, the first author, W.A.S., who had no professional or private relation with the HCP at the time of the interviews, carried out both the recruitment and data collection. All data was de-identified and preliminarily coded before L.A. was introduced to them. In this study, the first author, W.A.S. is a researcher and specialized nurse with qualitative research experience but is inexperienced with the patient group. She had previously worked as a coordinator of a specialized hospital team that assessed children with neurological symptoms, where one of the pediatricians and one physical therapist also worked. The strategy to handle the previous professional relationship was to stay focused on the topic of this study from the beginning to the end of the interviews. Further, to analyze the data from the interviews from de-identified transcripts. There was no contact between the researcher and the participants during the analysis. The last author (L.A.) is a physical therapist with clinical experience from neurodevelopmental follow-up. L.A. also has quantitative research experience in all three participating hospital sites through the implementation study, but not in ordinary clinical follow-up activities. To reduce the risk of influence on data collection and interpretation, L.A. was not involved in recruitment or interviews and was only included in analyses of de-identified and preliminary coded data. The authors discussed the possibility of bias and the need to be aware of and not discuss preliminary results with the participants during analyses. W.A.S. led the analysis process and had a critical view regarding potential bias throughout the research process.

The authors W.A.S. and L.A. developed the interview guide (Table 1). L.A. introduced the content of the remote GMA project and suggested themes for the interview guide. W.A.S. commented on the themes and suggested several open questions that could capture the essence of the experiences of interest. The interview guide was discussed several times before a final version was agreed upon by W.A.S. and L.A. Questions were adapted to the individual interviews and to the focus group interview. The interview guide aimed to collect data about how the GMA results were communicated in medical journals, to HCP, and to parents, and how they influenced clinical follow-up. The interview guide also aimed to collect data on experiences with carrying out the video recording of infant spontaneous movements in the hospital outpatient clinic.

Two individual interviews were conducted face-to-face in a meeting room at one of the hospitals. The focus group and two other individual interviews used a secure digital platform. W.A.S. interviewed all HCP. All interviews were audio-recorded and transcribed. Each HCP gave one interview. The individual interviews lasted for 26 to 56 min, and the focus group lasted for 55 min. The total length of all transcripts was 61 pages. While the longest interviews went deeper into the responses, all interviews confirmed findings. For example, when the researcher added questions based on information from previous interviews. Several of the participants shared similar experiences, which indicated that saturation was assessed.

The data analyses were inspired by Malterud’s Systematic Text Condensation (STC) [13]. This cross-case analysis strategy involves a four-step analysis procedure and is developed from Giorgis’ psychological phenomenological analysis. Step 1 included reading the transcript to gain an overall impression of the data and identify preliminary themes. In this step, it was noticed that pediatricians focused on missing details from GMA more than other participants. However, other participants also mentioned that pediatricians missed details from GMA. In step 2, codes were developed from meaningful units in the transcripts. In step 3, the meaningful units were systematically abstracted by writing a condensate, maintaining the HCPs’ phrasings. In step 4, the codes and condensate were discussed with L.A. before they were transformed by W.A.S. into the analytical text. Quotes were added to emphasize the findings. The findings were checked against the transcripts repeatedly for validation. This action confirmed that the final themes were derived from all interviews. The analysis process is illustrated in Table 2. Mind Manager version 24.0.181 was used to systematize the data. All procedures were carried out with standards approved by the Regional Ethical Committee in Mid Norway (62240).

## 3. Results

The findings from the interviews describe experiences with remote general movement assessment (GMA) to predict risk for cerebral palsy (CP) in high-risk infants, regardless of whether video recordings were performed at home or at the hospital. Eight health care personnel (HCP) who were engaged in local follow-up and the use of remote GMA participated with their experiences. The findings are presented in the following themes: GMA is an important examination contributing to earlier initiation of correct follow-up actions and appropriate treatment. Pediatricians wanted more details about the spontaneous movements’ qualities that could support clinical practice. A gentle examination—hospital video recording experiences, and parents were given an active role with home video recording.

### 3.1. GMA Is an Important Examination Contributing to the Earlier Initiation of Correct Follow-Up Actions and Appropriate Treatment

All HCPs talked about the GMA method as an important assessment tool that identified adverse development in young infants. Absence of the fidgety type of general movements (FM-) with GMA was perceived as an important indicator of high risk for CP. The focus group members agreed that: “We have faith in the GMA method. It confirms or disproves suspicion of CP in a gentle way”.

It was emphasized that the GMA method supported current clinical practice and was used together with several other assessment tools to consider risk for CP. The infants were already referred to the local hospital’s outpatient follow-up program due to medical risk factors for adverse development. Follow-up was initiated according to the standardized neurodevelopmental follow-up program and maintained as scheduled. Hence, providing GMA results, whether they revealed low risk (FM+) or high risk (FM-) for CP, did not change the routine and frequencies of hospital-based follow-up consultations.

Sometimes, the clinical interpretation of GMA results could be uncertain, like an FM sporadic or exaggerated FM classification. One extra GMA video recording was useful for verification of results, and further, contact was often established with a local municipality’s physical therapist, which usually resulted in closer local follow-up.

A positive consequence of the GMA results could be earlier initiation of correct local follow-up actions and appropriate treatment. For example, services, such as the habilitation team and involvement of the local municipality health care, were approached if GMA showed “at risk for CP”. Thus, infants at risk could be offered a closer and individualized follow-up in the local municipality. The experience among HCP in this study was that HCP in municipalities responded quickly. Local actions for the infants and families were initiated if concerns were raised by the hospital follow-up team based on their professional understanding of the GMA results. A physical therapist stated that “You perceive that the local municipality HCP understand the seriousness of the situation and that the infant usually receives more interdisciplinary follow-up quite quickly.”

Another important aspect was that the parents were given explanations as to why closer follow-up was desirable if GMA results indicated “at risk for CP”. The HCP expressed that the parents’ understanding of the situation communicated through the GMA results could contribute to improved cooperation concerning their infants. In contrast, if fidgety movements were observed (FM+) and this was in line with other results reported from the follow-up team, parents could be reassured that the infant had a low risk for CP.

### 3.2. Pediatricians Wanted More Details About the Spontaneous Movement Qualities That Could Support Clinical Practice

When GMA demonstrated intermittent or continuous presence of fidgety movements, the conclusion was that the child had “low risk for CP”. That was perceived as an important contribution by the GMA. A pediatrician expressed that “When the map and terrain match, it’s very good, and the times when we are a little more unsure of what the result means or maybe a little surprised by the result, we become more uncertain.”

Some of the pediatricians expressed the wish to have a more detailed description of the GMA results. The focus on FMs only and not on concurrent motor repertoire could lead pediatricians to wonder whether there were any nuances in the GMA that were not communicated. Especially in cases when different video recordings of the same individual infant showed different GMA results. This could create uncertainty as to whether there were results that had not been communicated to the pediatrician. Therefore, it was mentioned that further studies should include information about the optimality of the concurrent motor repertoire, in addition to the analysis of FMs. For example, a description of normal and abnormal concurrent motor repertoires could be useful. Also, in the case of abnormal movement patterns, in what way was it considered that the right or left, lower or upper extremities were affected?

### 3.3. A Gentle Examination—Hospital Video Recordings

However, the HCP had reflected upon the ethical aspect of video recording an infant in a vulnerable position, only dressed in a nappy or with a onesie lying on the floor. Several of the HCP mentioned a potentially traumatic situation and that the infant could feel that those who recorded the video had the upper hand. Having to withhold a response to the infant’s attempt for attention during a video recording could be a challenge. A second physical therapist said that: “During three minutes of video recording when we are not supposed to say or do anything—it is a still face experiment”.

Some infants could have difficulties lying alone in the supine position, as well as regulating themselves. At the same time, parents could have challenges putting the infants down and leaving them alone on a mattress. The mothers would naturally want to comfort the infant if they made noises for attention. However, the HCP found it amazing to watch the infants recover, how resilient they were, and that they turned into smiling and babbling when the video recording was over.

### 3.4. Parents Were Given an Active Role with Home Video Recording

The parents also performed video recordings themselves at home and conveyed to HCP that it was experienced as a positive activity. The HCP said that a first-time video recording at the hospital could improve the parents’ video recording at home. For instance, the parents could benefit from observing and experiencing that they did not have to comfort the infant every time there was a sound. HCP believed that parents managed home-video recording well and that the quality of the home-based videos was good. Simultaneously, parents were given an active role where they could experience video recording as a safe examination for the child. HCP experienced that the parents wanted to assist their infant in the best possible way.

Reducing travel activities to the hospital for video recording could reduce the burden and stress for both the infant and parents as a consequence of home video recording. At home, the infant was in a safe and comfortable setting and could be video recorded when it was most convenient. Parents could upload the most optimal video out of several to the hospital. The HCP expressed that “Home video recording is resource-saving in every way”.

## 4. Discussion

This study describes health care personnels’ (HCPs’) experiences with remote general movement assessment (GMA) for early detection of cerebral palsy (CP) in infants between 12 and 17 weeks post-term age. The experiences were based upon hospital and home-based video recordings, and how the GMA results influenced the HCP’s clinical decisions about further follow-up actions in a Norwegian high-risk infant cohort.

HCPs in local follow-up programs experienced that GMA was a gentle way to assess infants’ risk of CP before 5 months post-term age. GMA was considered a useful supplement to existing assessment tools in decision-making for further follow-up. The hospital follow-up team experienced communication of GMA results to the municipality. HCPs improved their understanding of the early risk for CP, and consequently initiated a closer follow-up if GMA had abnormal results. However, pediatricians in the hospital follow-up team sometimes could miss a more detailed description of the quality of infant motor repertoires that could support their clinical decisions and actions for further follow-up at the hospital. Ethical reflections concerning video recording performed by HCPs at the hospital outpatient clinic revealed that it was a potentially stressful situation for the infant, but also that the infants were resilient and recovered quickly after the recording session. The parents were given an active role in performing home-based videos, which were considered resource-saving in different ways.

Our findings on HCPs’ positive experiences with remote GMA support what has previously been reported on parental experiences with home-video recording [11]. The experiences among HCPs, that parents can easily perform home video recording, are also in line with what parents themselves report [8,11]. In addition, parents reported in the Adde et al. study that being involved in home video recording made them more attentive towards their infant’s development [8]. This might be a positive basis for what was found in our study, that HCPs experienced the use of GMA as helpful in dialogs with parents about further follow-up steps. However, Kukka et al. previously described a need for technical support among Nepalese mothers to be self-confident in using smartphone apps for remote GMA [12]. A study by Grimshaw et al. found that a successful translation of knowledge is dependent on the identification of local barriers [14]. The positive experiences with GMA in this study might be because the HCPs worked in hospital sites that had already partly adapted to the implementation of GMA locally.

The feasibility of the implementation of early at-risk diagnosis for CP has previously been demonstrated [15,16], and our study supports these findings from the perspective of HCPs in follow-up programs of high-risk infants. Our finding that interventions, thus, could be initiated earlier is also described by Byrnes et al. [15]. HCPs in our study seem to have the same priority [15], optimizing early clarification and planning of follow-up steps to come.

Receiving an early diagnosis or high-risk for CP classification is also found to be a priority for parents [11,12,15]. Also, Kukka et al. describe that Nepalese mothers’ perspectives are that they hope remote GMA will benefit their children [12]. However, awareness is needed toward cultural differences regarding home video recording, i.e., if parents have a perception that the videos might be used for other purposes than remote GMA [12].

The ethical concerns raised by participants in our study regarding distress during video recordings in hospitals and challenges for parents withholding comfort are to the best of our knowledge, new and previously not described in the literature. How these issues could impact the implementation of GMA in other cultural or health care contexts could be important to explore in the future.

Pediatricians wished for a more detailed description of the quality of infant motor repertoire. A previous study by Sermpon & Gima [17] has reported that the frequency of movement toward midline (MTM) was associated with the presence of fidgety movements. Further, a study by Kihara et al. [18] demonstrated that a set of broader motor features predicted later neurodevelopmental outcomes beyond GMA. Finally, a study by Gima et al. [19] has identified early motor signs of autism spectrum disorders related to head movement patterns. Hence, such avenues for other qualitative and quantitative markers have the potential to complement GMA with more detailed descriptions in early screening.

Strengths and limitations: This study used a small, predefined sample of HCPs who had experience from a defined clinical study with early detection of risk for CP in infants using remote GMA. Most of the invited HCP chose to participate, which was a strength, since exploring their experiences was the aim of the study.

The HCP represented clinical professionals who are engaged in the usual local follow-up teams in hospitals assessing infants for risk of CP. This was considered a strength, since their experiences reflect their true field of daily work. It was also a strength that HCPs were recruited from three different hospitals, giving a variability of contexts in follow-up programs. HCP from one of the hospitals were trained GMA observers, while HCP from the two other hospital sites did not have GMA-specific knowledge besides general knowledge from literature. This probably reflects the general HCP’s knowledge in local hospital follow-up teams, with no GMA expertise about early risk assessment for CP.

The sample was appropriate for a qualitative study, and the participants’ experiences may be transferable to similar contexts. However, the trained GMA observers in one of the sites could also represent a limitation, since they could have promoted GMA through ordinary interdisciplinary dialogs within a health region. In addition, the HCPs were recruited from an ongoing study where GMA was implemented, which may have influenced their attitudes and trust towards the use of GMA. Due to these factors, a possible limitation could be that the HCPs had faith and a positive preconception, in general, in relation to early identification of the risk of CP using the GMA method. This might have impacted the responses in this study, leaving out important critical views. This may also explain the overwhelmingly positive experiences reported. The lack of dissenting voices might be a limitation of our study.

It was a possible limitation of this study that one of the authors was familiar with and had responsibilities in the GMA implementation study from which HCPs were recruited. However, it can be argued that the fact that the first author who recruited HCP, collected and led the data analysis, and was unfamiliar with the patient group and most of the HCP, reduced this risk. This also promoted critical reflections during the research process. The findings were checked repeatedly against the transcripts to ensure that the HCP’s voices were reflected.

## 5. Conclusions

In the current study, health care personnel (HCP) working in local hospital follow-up programs for high-risk infants experienced that general movement assessment (GMA) was a gentle way to assess the risk of cerebral palsy (CP) between 12 and 17 weeks post-term age. Communication with parents and the local municipality HCP improved based on the GMA results and led to a closer municipality follow-up if GMA had abnormal results. A more detailed assessment of infant motor behaviors might be beneficial in addition to the assessment of FMs to support clinical decisions and provide even more individualized follow-up planning. Thus, remote GMA might be able to support more developmental issues than the risk of CP. These insights may be of value to similar implementation projects.

## Figures and Tables

**Table 1:** Questions from the interview guide (translated from Norwegian).

Can you tell us about your experiences of finding and reading general movement assessment (GMA) results from the medical journal?
How have you experienced that the GMA results have been communicated in the medical journal?
How do you think GMA results should be described and communicated?
Have you missed something in the description of the GMA results?
In what way have the GMA results been important in relation to the follow-up of patients?—at high/low risk
In what way have the GMA results been important in relation to examinations of the child?—at high/low risk (more or fewer examinations?)
What practical consequences have the GMA results had in relation to the assessment and follow-up of children and parents?
In what way have the GMA results been communicated to parents?
How have you experienced communicating GMA results to parents?
How have you experienced that parents have reacted to the results of the early risk assessment of the child?
Can you tell us about your experiences with conducting video recordings in the GMA study?
What do you think about video recordings of the child being made at home?
What do you think about video recordings of the child being made at the hospital?
Is there anything else you would like to share about your experiences with GMA?

**Table 2:** Examples from the analysis inspired by Malterud’s STC four steps [13].

Step 1 Preliminary Themes	Step 2 Meaningful Units Were Coded (Highlighted and Marked with Grey) and Grouped in Themes. From This the Condensate Was Written.	Step 3 From Condensed Meaning of the Phrasings we Wrote the Analytical Text	Step 4 Final Themes (Quotes Were Added)
Earlier diagnosis Initiating support Same understanding Better cooperation	“I would say that it has led to those who do not have fidgety movements (…) it still contributes to making an **earlier diagnosis** and also **initiating the support system** around the individual.” “The method helps to **detect deviations earlier** than if we had not used it” “Not necessarily additional examinations, … it is said that **the risk of cerebral palsy** when you do not have fidgety movements is quite high, and parents are also informed about this.” “(…) **we are already concerned about the child** and have thought about and initiated measures.” **“I don’t think anyone gets more or less than what was initially thought**. (…) **you get confirmation or refutation of the suspicions** you have had (…) in a very gentle way, since it is still so easy to perform that assessment.” …. “(…) **it is very helpful for parents** to be as concerned as we are. (…) we have **the same perception, and it makes it easier** (…) **it is easier to cooperate when you understand the same thing**. (…) if we also have those who have a low risk of getting CP, it is also pleasing.” “It is precisely the contact and understanding you get with the parents when you present the result that is perhaps the big change. **You get the same focus and they get a different understanding** (…). I think that is the big positive part of it.”	Absence of the fidgety type of general movements (FM-) with GMA was perceived as an important indicator of high risk of CP. It was emphasized that the GMA method supported current clinical practice and was used together with several other assessment tools to consider risk for CP. The infants were already referred to the hospital’s outpatient follow-up program due to medical risk factors for adverse development. Follow-up was initiated according to the neurodevelopmental follow-up program and maintained as scheduled. Hence, providing GMA findings, whether they revealed low risk (FM+) or high risk (FM-) for CP, did not change the routine and frequencies of hospital-based follow-up consultations … Another important aspect was that the parents were given explanations as to why closer follow-up was desirable if GMA findings indicated “at risk for CP”. The participants expressed that the parents’ understanding of the situation communicated through the GMA findings could contribute to improved cooperation concerning their infants. In contrast, if fidgety movements were observed (FM+) and this was in line with other findings from the follow-up team, parents could be reassured that the infants had a low risk for CP.	GMA is an important examination contributing to earlier initiation of correct follow-up actions and appropriate treatment
Prepared for the video recording Avoid disturbances like sounds and colors	“(…) **they have received very good information about what will happen during the testing or filming.** “(…) **they are well prepared** because the parents are informed in advance (…).” “We’ve learned a bit from our mistakes… (…) there’s **a box with lots of balls, nice colors — we found out that wasn’t such a good idea.**,(…), **we’ve started placing the child with the back of their head facing the ball pit**.”	The parents were well informed in advance about the setting up of GMA, and thus, were well prepared for the video recording of the infant at the hospital. It was important to ensure that external factors that could influence the infant state and movements, like visual stimulation or sounds, were not present and did not disturb the infant.	A gentle examination—hospital video recording experiences

## Data Availability

Data is available upon reasonable request. The transcripts of the in-depth interviews underlying the findings presented in this study are not publicly available due to regulations of the Regional Ethical Committee for medical and health research in Norway. The anonymity of the informants must be secured. In the raw data, it is possible to identify the informants, and restrictions therefore apply to the availability of these data. Reasonable requests concerning the data can be sent to the corresponding author.

## Abbreviations

The following abbreviations are used in this manuscript:

GMA	General movement assessment
CP	Cerebral palsy
HCP	Health care personnel
FM	Fidgety type of infant, general movement
STC	Systematic Text Condensation
FM+	Low risk of cerebral palsy
FM−	High risk of cerebral palsy
